# Stroke Care Core Competencies Among Nurses at Mbarara Regional Referral Hospital, Southwestern Uganda

**DOI:** 10.7759/cureus.109900

**Published:** 2026-05-29

**Authors:** Josephine N Najjuma, Scovia Nalugo Mbalinda, Frank Ssedyabane, Mark Kaddumukasa, Shifra Nankunda, Esther C Atukunda

**Affiliations:** 1 Nursing, Mbarara University of Science and Technology, Mbarara, UGA; 2 Department of Nursing, School of Health Sciences, College of Health Sciences, Makerere University, Kampala, UGA; 3 Medical Laboratory Science, Mbarara University of Science and Technology, Mbarara, UGA; 4 Department of Internal Medicine, Makerere University, Kampala, UGA; 5 Pharmacy, Mbarara University of Science and Technology, Mbarara, UGA

**Keywords:** competency-based education, needs assessment, nursing competencies, stroke, uganda

## Abstract

Background: Nurses play a key role in stroke patient care in hospitals, yet little is known about their stroke-specific competencies in low-resource settings like Uganda. Assessment of stroke care competency among new and experienced nurses is important for identifying areas of improvement and development of professional programs to enhance the quality of care for patients. This study assessed self-reported stroke care competencies among nurses at Mbarara Regional Referral Hospital (MRRH) to identify priority training needs.

Methods: We conducted a cross-sectional descriptive study at MRRH, from September to November 2025, using the Stroke Core Competency Framework (SCCF), a self-assessment tool with 24 competencies based on Benner's Novice-to-Expert scale. All nurses caring for stroke patients (N=25) completed a paper-based questionnaire. Data was analyzed in Stata, release 17 (StataCorp LLC, College Station, USA).

Results: A total of 25 nurses, with a mean age of 31.8 (SD ±7.2) years, were enrolled; 52% (13/25) were female participants. The majority of the participants had attained diploma training (12/25, 48%), and only 8% (2/25) had more than 15 years of experience in nursing care. For most of the competencies and the corresponding items, most rated themselves as novice or advanced beginners across domains (e.g., 16/25 (64%) in activity/mobilization; 16/25 (64%) in dysphagia assessment), with higher competence in basic anatomy/physiology.

Conclusion: The majority of nurses are either novice or advanced beginners when it comes to competence levels for stroke care. Context-specific and competency-based continuing education is needed in implementing stroke evidence-based practices and designing stroke-based training for nurses to achieve optimal competency.

## Introduction

Stroke is a devastating condition that is reported as the second leading cause of mortality and long-term disability worldwide [1]. The incidence of stroke is projected to increase because of the aging population [2], although in low- and middle-income countries (LMICs), more young people are affected by stroke due to increased prevalence of stroke risk factors among other causes [2,3]. It is also estimated that LMICs contribute 85% of the global burden of incident intracerebral hemorrhage [4,5]. In Uganda, stroke accounts for 15% of hospital admissions and constitutes a major contributor to mortality. It has been reported that in southwestern Uganda, the overall 30-day stroke mortality is 38.1% [6].

Stroke patients and survivors have complex needs that require the expertise and clinical skills provided by well-trained healthcare professionals. Healthcare providers play a vital role in stroke care, treatment and prevention of complications of stroke among survivors, and prevention of secondary stroke [7]. For this, it is recommended that healthcare providers receive education and training to deliver evidence-based stroke care [8,9]. Nurses offer a significant contribution to care for stroke patients and acute stroke treatment, preventing patient deterioration and enhancing rehabilitation potential and outcome [10,11]. For nurses to deliver patient-centered care, work in interdisciplinary teams, employ evidence-based practice, apply quality improvement, and utilize informatics, they need to have a strong set of core competencies. Interventions to improve stroke outcomes are geared toward improving nurses’ competencies in stroke care [12].

Nurses working with stroke patients require specialist training and development of competencies in dealing with neurological emergencies and working in multidisciplinary teams [13]. The crucial role of nurses in stroke care requires an understanding of different stroke care competencies [11] that may include understanding the pathology of stroke and the provision of the required care [14]. Development of these training packages should be informed by the current level of the nurses’ competencies in stroke care. The Stroke Core Competency Framework (SCCF) assesses the entire continuum of stroke care to identify the learning needs of healthcare providers after self-assessment [15]. In this study, we assessed baseline stroke core competencies among nurses caring for stroke patients at a regional referral hospital in southwestern Uganda to identify priority training needs.

## Materials and methods

Study design and setting

We carried out a cross-sectional descriptive study from September to November 2025 to describe baseline stroke core competencies among nurses caring for stroke patients. The study was conducted at Mbarara Regional Referral Hospital (MRRH) in southwestern Uganda, which admits approximately 25 stroke patients per month through the emergency ward and surgical and medical sections. Following medical evaluation, they are later transferred to the ICU, medical ward, or surgical ward, depending on the type of stroke, severity of disease, and type of intervention planned. The care offered to patients with stroke is managed by multidisciplinary healthcare professionals, including physicians, neurosurgeons, medical officers, nurses, physiotherapists, and radiologists, though nurses make up the largest proportion of the healthcare workforce. The hospital serves the 15 districts in southwestern Uganda and neighboring countries, including Tanzania, Rwanda, and the Democratic Republic of the Congo (DRC).

Participants

We attempted to conduct a census and included all nurses who offer care to stroke patients in all wards of the hospital. These included nurses from the ICU, emergency ward, medical ward, and surgical ward. These nurses had spent at least two months taking care of stroke patients or rotating on a ward where stroke patients are admitted. All nurses who met the inclusion criteria were invited to participate in the study (30 nurses met the inclusion criteria, but 25 participated).

Stroke Core Competency Framework

The SCCF was used to assess the nurses’ competencies. It is a self-assessment tool for healthcare providers that is used to identify key skills and knowledge needed for evidence-based stroke care [15]. It covers competencies from acute care to rehabilitation and helps with orienting staff, professional development, and performance reviews. It was rebranded by the developers from the Provincial Interprofessional Stroke Core Competence Framework in 2022 and included 24 competencies in stroke care. The SCCF is designed for use by both new and experienced healthcare providers working across the continuum of stroke care and uses Benner’s Novice-to-Expert model [16]. Benner's stages of clinical competence include novice, advance beginner, competent, proficient, and expert. 

A group of six experts (three stroke care nurses and three educators) verified the content validity of the instrument using the content validity index (CVI). This is because the study population was different from Canada, where it was first verified. According to Artino et al., a group of 6-10 experts should be consulted [17]; the items that scored 0.78 and higher were considered in the final tool. These satisfied the recommended guideline by Polit and Beck [18].

Data collection

We used a paper-based self-administered tool to collect the data. The SCCF was used to rate the competencies across the stroke core continuum. The nurses’ demographic characteristics included age, sex, level of education, ward, and years of experience. The tool was administered in English since all nurses were able to read and rate in English, as English is the official language of Uganda. All participants were individually contacted by a trained research assistant who explained the overview and purpose of the study. The research assistant also shared with them the consent forms and obtained written informed consent after answering all the questions from the participants. Data was collected from September 2025 to November 2025.

Data analysis

Data was analyzed using Stata, release 17 (StataCorp LLC, College Station, USA). We summarized categorical data using frequencies and proportions, while numerical data was presented using means and standard deviations.

Ethical considerations

This study was approved by the Research Ethics Committee, Mbarara University of Science and Technology (MUST-2023-1175) and the Uganda National Council for Science and Technology (HS3535ES). All participants provided written informed consent after receiving a detailed explanation of the purpose of the study, participants' rights to confidentiality, autonomy to withdraw, and procedures of the study. Each participant was given a unique identifier that was used for identification instead of their names. Only participants' unique identification numbers were included in the dataset. All study data was collected and kept in a password-protected computer that was only accessed by the study team.

## Results

Study participants included nurses from all four wards at MRRH, where stroke patients receive care. The mean age of the participants was 31.8 (±7.2) years. Barely half of the participants were female (13/25, 52%); close to half of the participants (12/25, 48%) were diploma holders, as shown in Table 1.

**Table 1:** Demographics of the participants

Variable	Category	Frequency (N=25), n (%)
Age (years), mean (±SD)		31.8 (7.2)
Gender	Female	13 (52)
	Male	12 (48)
Years of experience	0-1	6 (24)
	1.1-5	6 (24)
	5.1-10	5 (20)
	10.1-15	6 (24)
	>15	2 (8)
Highest level of education	Certificate	7 (28)
	Degree	6 (24)
	Diploma	12 (48)

Anatomy, physiology, and hyperacute care for stroke

For the Introduction to stroke care competency, the majority of participants rated themselves competent to expert, with a higher expert rating for defining stroke rehabilitation. For Brain anatomy and physiology and Stroke pathophysiology, the majority of the participants rated themselves as advanced beginners or competent. For the domain item Describe how neuroplasticity affects stroke recovery, more participants rated themselves novice, and it was identified as a learning need for the nurses. For the Hyperacute stroke care competency, the majority of the participants rated themselves as competent or below. There were more advanced beginners for the following items: Describe the role of Emergency Medical Services in the management of acute stroke (12/25, 48%), Explain care processes for Transient Ischemic Attack management in the emergency department (13/25, 52%), Identify stroke complications during the hyperacute phase (11/25, 44%), Describe interventions to prevent and manage hyperacute stroke complications (10/25, 40%), and Select and complete the most appropriate neurological assessment (12/25, 48%), as shown in Table 2.

**Table 2:** Ratings on anatomy, physiology and hyperacute care for stroke

Competency	Domain items	Frequency (proportion), N=25				
		Novice, n (%)	Advanced beginner, n (%)	Competent, n (%)	Proficient, n (%)	Expert, n (%)
Introduction to stroke care	Demonstrate respect for cultural differences and values	1 (4)	7 (28)	11 (44)	4 (16)	2 (8)
	Describe the roles and scope of practice	2 (8)	5 (20)	6 (24)	6 (24)	5 (20)
	Recognize the value of interprofessional collaboration	3 (12)	6 (24)	9 (36)	4 (16)	3 (12)
	Define stroke rehabilitation	3 (12)	5 (20)	7 (28)	3 (12)	6 (24)
Brain anatomy and physiology	List the anatomical structures of the brain	1 (4)	4 (16)	14 (58)	3 (12)	2 (8)
	Describe the functions of each major area of the brain	1 (4)	8 (32)	12 (48)	3 (12)	0 (0)
	Identify the major arteries of the cerebrovascular circulation	4 (16)	7 (28)	11 (44)	2 (8)	1 (4)
	Describe how neuroplasticity affects stroke recovery	5 (20)	11 (44)	6 (24)	2 (8)	1 (4)
Stroke pathophysiology	Explain the pathophysiology of ischemic and hemorrhagic stroke	2(8)	9 (36)	9 (36)	3 (12)	2 (8)
	Describe the etiologies of ischemic and hemorrhagic stroke	1 (4)	8 (32)	9 (36)	5 (20)	2 (8)
	Describe clinical impairments associated with major stroke syndromes	3 (12)	6 (24)	6 (24)	7 (28)	2 (8)
Hyperacute stroke care	Describe the role of Emergency Medical Services in the management of acute stroke	0 (0)	12 (48)	8 (32)	4 (16)	1 (4)
	Recognize signs of stroke and how to respond appropriately based on your role	1 (4)	3 (12)	14 (56)	4 (16)	3 (12)
	Explain the importance of timely assessment and tools to promote rapid identification of an acute stroke	1 (4)	5 (20)	13 (52)	2 (8)	4 (16)
	Explain care processes for Transient Ischemic Attack management in the emergency department	4 (16)	13 (52)	5 (20)	3 (12)	0 (0)
	Describe hyperacute stroke therapies	5 (20)	9 (36)	9 (36)	2 (8)	0 (0)
	Identify stroke complications during the hyperacute phase	4 (16)	11 (44)	8 (32)	1 (4)	1 (4)
	Describe interventions to prevent and manage hyperacute stroke complications	3 (12)	10 (40)	9 (36)	2 (8)	0 (0)
	Select and complete the most appropriate neurological assessment	2 (8)	12 (48)	8 (32)	2 (8)	1 (4)
	Manage hyperacute stroke care and associated complications	4 (16)	9 (36)	10 (40)	2 (8)	0 (0)
	Effectively communicate the recommendations	3 (12)	8 (32)	10 (40)	2 (8)	1 (4)

Stroke types, dysphagia, and nutrition and hydration for persons with stroke

Most participants rated themselves as advanced beginners in most of the domains, although in some domains, the participants rated themselves as competent. For the Dysphagia competency, for Explain dysphagia and the purpose of dysphagia screening tool prior to initiating oral intake, 10 of 25 (40%) rated themselves as advanced beginners, and for Complete the assessment for dysphagia (swallow study, evaluation of swallowing), 10 (40%) rated themselves as advanced beginners. For the Nutrition and hydration competency, for the two items that were related to malnutrition, close to half of the participants rated themselves as advanced beginners (10/25, 40%), as shown in Table 3.

**Table 3:** Ratings on stroke types, dysphagia, and nutrition and hydration for persons with stroke

Competency	Domain items	Frequency (proportion), N=25				
		Novice, n (%)	Advanced beginner, n (%)	Competent, n (%)	Proficient, n (%)	Expert, n (%)
Ischemic and intracerebral hemorrhage stroke care	Describe acute stroke care assessment and management for ischemic and intracerebral hemorrhagic strokes	1 (4)	8 (32)	14 (56)	2 (8)	0 (0)
	Describe the diagnostic tests used to support stroke diagnosis and treatment	4 (16)	8 (32)	8 (32)	4 (16)	1 (4)
	Identify possible post-stroke complications during the acute phase	2 (8)	7 (28)	11 (44)	4 (16)	1 (4)
	Describe interventions to prevent and manage complications	1 (4)	7 (28)	12 (48)	3 (12)	1 (4)
Dysphagia	Explain dysphagia and the purpose of dysphagia screening tool prior to initiating oral intake	1 (4)	10 (40)	11 (44)	3 (12)	0 (0)
	Describe signs and symptoms of swallowing difficulties and associated complications	1 (4)	7 (28)	11 (44)	5 (20)	1 (4)
	Demonstrate proper positioning and setup for safe feeding	1 (4)	7 (28)	9 (36)	4 (16)	4 (16)
	Explain the connection between dysphagia deficits and location of the stroke	6 (24)	8 (32)	10 (40)	1 (4)	0 (0)
	Complete the assessment for dysphagia (swallow study, evaluation of swallowing)	6 (24)	10 (40)	8 (32)	1 (4)	0 (0)
	Interpret assessment results and provide appropriate recommendations	3 (12)	7 (28)	12 (48)	2 (8)	1 (4)
	Manage dysphagia and their associated complications	2 (8)	6 (24)	10 (40)	5 (20)	2 (8)
	Explain the factors to consider when making decisions regarding tube feeding	1 (4)	8 (32)	12 (48)	4 (32)	0 (0)
	Effectively communicate the recommendations to the persons with stroke, families/caregiver and the interprofessional team	1 (4)	7 (28)	9 (36)	6 (24)	2 (8)
Nutrition and hydration	Describe the importance of screening for nutrition and hydration	1 (4)	7 (28)	7 (28)	4 (16)	6 (24)
	Screen for malnutrition using a validated screening tool, know when to re-assess and consult	4 (16)	6 (24)	9 (36)	3 (12)	2 (8)
	Describe the various diets, textures, and alternative feeding methods	5 (20)	3 (12)	8 (32)	5 (20)	4 (16)
	Demonstrate proper positioning and setup for safe feeding	2 (8)	7 (28)	6 (24)	7 (28)	3 (12)
	Describe the sequelae of malnutrition	4 (16)	10 (40)	7 (28)	2 (8)	1 (4)
	Identify persons with stroke at risk for malnutrition	2 (8)	10 (40)	6 (24)	6 (24)	1 (4)

Oral care, activity, and communication

For the Oral care competency, most of the participants were advanced beginners. For the Activity post-stroke and safe patient handling competency, more than half were novice or advanced beginners for the following four items: Identify contraindications to activity and mobilization (14/25, 56%), Identify fall prevention strategies (15/25, 60%), Describe best practice recommendations for the upper and lower extremity (16/25, 64%), and Select and complete the most appropriate assessment(s) to evaluate function and safety (16/25, 64%). For the Changes in communication competency, more than half of the participants were advanced beginners for all three items, as shown in Table 4.

**Table 4:** Ratings on oral care, activity and communication

Competency	Domain item	Frequency (proportion), N=25				
		Novice, n (%)	Advanced beginner, n (%)	Competent, n (%)	Proficient, n (%)	Expert, n (%)
Oral care	Identify sequelae of poor oral care and prevention strategies	1 (4)	5 (20)	5 (20)	5 (20)	3 (12)
	Screen oral health and perform oral care according to your organizational protocol	4 (16)	10 (40)	5 (20)	3 (12)	3 (12)
Activity post-stroke and safe patient handling	Explain the role of the interprofessional team in supporting activity and	2 (8)	6 (24)	8 (32)	7 (28)	2 (8)
	Describe the importance of early activity and mobilization in stroke	2 (8)	7 (28)	7 (28)	7 (28)	2 (8)
	Describe how muscle tone, posture, balance, muscle weakness/motor loss, sensory loss, and fatigue can affect function and safe patient handling	0 (0)	12 (48)	9 (36)	1 (4)	3 (12)
	Demonstrate proper techniques for handling, positioning, and transferring	2 (8)	8 (32)	10 (40)	3 (12)	2 (8)
	Demonstrate proper use of appropriate equipment or devices to facilitate activity	4 (16)	8 (32)	10 (40)	1 (4)	1 (4)
	Identify contraindications to activity and mobilization	3 (12)	11 (44)	6 (24)	4 (16)	1 (4)
	Identify fall prevention strategies	4 (16)	11 (44)	4 (16)	4 (16)	2 (8)
	Describe best practice recommendations for the upper and lower extremity	4 (16)	12 (48)	6 (24)	3 (12)	0 (0)
	Select and complete the most appropriate assessment(s) to evaluate function and safety	3 (12)	13 (52)	5 (20)	4 (16)	0 (0)
Changes in communication	Explain the implications of communication impairments on participation and engagement in therapy and activity	3 (12)	14 (56)	5 (20)	2 (9)	1 (4)
	Demonstrate strategies to improve or assist with communication when interacting with those who have communication deficits to assist with participation in conversation, following instructions and informed decision making	6 (24)	11 (44)	7 (28)	0 (0)	1 (4)
	Describe the impact of the environment on communication and provide strategies to maximize successful communicative interactions	5 (20)	14 (56)	4 (16)	1 (4)	1 (4)

Post-stroke changes in body physiology

For all five competencies related to changes in physiology post-stroke, more than half of the participants rated themselves as advanced beginners, followed by those who rated themselves as competent (Table 5).

**Table 5:** Ratings on changes in body physiology post-stroke

Competency	Domain items	Frequency (proportion), N=25				
		Novice, n (%)	Advanced beginner, n (%)	Competent, n (%)	Proficient, n (%)	Expert, n (%)
Changes in cognition	Identify common cognitive changes post-stroke	2 (8)	12 (48)	6 (24)	5 (20)	0 (0)
	Describe the impact of cognitive changes on function	6 (24)	10 (40)	6 (24)	2 (8)	1 (4)
	Understand the relationship between mood and cognitive changes post-stroke	7 (28)	10 (40)	5 (20)	3 (12)	0 (0)
	Understand the impact of the environment on cognitive function post-stroke	7 (28)	7 (28)	4 (16)	7 (28)	0 (0)
	Demonstrate strategies to optimize participation in functional activities	8 (32)	10 (40)	5 (20)	1 (4)	1 (4)
	Describe the clinical consideration for screening and assessing cognitive	6 (24)	11 (44)	6 (24)	1 (4)	1 (4)
Changes in vision and perception	Identify common visual and perceptual changes post-stroke	4 (16)	9 (36)	8 (32)	4 (16)	0 (0)
	Describe the impact of visual and perceptual changes on function	6 (24)	10 (40)	4 (16	3 (12)	2 (8)
	Understand the impact of the environment on visual-perceptual function	6 (24)	10 (40)	6 (24)	2 (8)	1 (4)
	Demonstrate strategies and interventions to optimize participation in functional activities when visual and perceptual deficits exist	5 (20)	10 (40)	8 (32)	1 (4)	1 (4)
Sensorimotor recovery	Demonstrate strategies and interventions to optimize motor and sensory	3 (12)	11 (44)	10 (40)	0 (0)	1 (4)
	Select and complete the most appropriate assessment(s) to enable sensorimotor recovery	7 (28)	10 (40)	7 (28)	1 (4)	0 (0)
	Interpret assessment results, identify/implement appropriate treatment	6 (24)	10 (40)	6 (24)	3 (12)	0 (0)
Fatigue and changes in mood and behavior	Describe post-stroke fatigue and provide strategies for self-management	4 (16)	10 (40)	7 (28)	3 (12)	1 (4)
	Describe common mood changes post-stroke and identify the warning signs	2 (8)	13 (52)	5 (20)	5 (20)	0 (0)
	Identify how stroke impacts behavior, emotions, actions and/or reactions	2 (8)	13 (52)	5 (20)	4 (16)	1 (4)
	Demonstrate strategies and interventions to manage the person’s mood	5 (20)	10 (40)	8 (32)	1 (4)	1 (4)

Physical function, continence, post-stroke pain, and spasticity

For all three competencies, most of the participants rated themselves as advanced beginners for the Changes in physical function competency. For item Understand the impact of the environment on physical function, more than half of the participants (15/25, 60%) rated themselves as novice or advanced beginners, and for Demonstrate strategies and interventions that optimize recovery, 16 (64%) rated themselves as novice or advanced beginners. For the Maintaining/promoting continence competency, for Apply tools and strategies to support toileting and continence, 60% (15/25) of the participants rated themselves as novice or advanced beginners. For Prevention and management of post-stroke pain and spasticity, most participants rated themselves as novice or advanced beginners (Table 6).

**Table 6:** Ratings on physical function, continence, post-stroke pain and spasticity

Competency	Domain items	Frequency (proportion), N=25				
		Novice, n (%)	Advanced beginner, n (%)	Competent, n (%)	Proficient, n (%)	Expert, n (%)
Changes in physical function	Identify physical changes that can occur post-stroke	4 (16)	8 (32)	9 (36)	1 (4)	3 (12)
	Describe the physical impacts of stroke on activities of daily living	4 (16)	8 (32)	8 (32)	2 (8)	3 (12)
	Understand the impact of the environment on physical function	5 (20)	10 (40)	6 (24)	2 (4)	2 (8)
	Demonstrate strategies and interventions that optimize recovery	5 (20)	11 (44)	6 (24)	1 (4)	2 (8)
Maintaining/promoting continence	Explain the loss of bladder (urinary retention and/or incontinence) and bowel control (fecal incontinence and/or constipation) and the impact on care needs of the person with stroke	3 (12)	9 (36)	6 (24)	4 (16)	3 (12)
	Describe risk factors for urinary incontinence/retention and fecal incontinence/constipation	2 (8)	6 (24)	10 (40)	3 (12)	4 (16)
	Apply tools and strategies to support toileting and continence	6 (24)	9 (36)	5 (20)	4 (16)	1 (4)
Prevention and management of post-stroke pain and spasticity	Describe complications seen in the hemiplegic arm	5 (20)	9 (36)	8 (32)	2 (8)	1 (4)
	Explain how altered tone affects persons with stroke	5 (20)	8 (32)	7 (28)	4 (16)	1 (4)
	Identify persons with stroke most at risk of developing spasticity	6 (16)	10 (40)	7 (28)	1 (4)	1 (4)
	Identify common types of post-stroke pain and describe recommended interventions	7 (28)	7 (28)	7 (28)	4 (4)	0 (0)
	Apply strategies to protect the hemiplegic arm and prevent injury	3 (12)	10 (40)	8 (32)	4 (16)	0 (0)
	Apply recommended positioning techniques, interventions, and tools to minimize pain and spasticity	7 (28)	6 (24)	7 (28)	4 (16)	1 (4)

Stroke prevention, transitions and community integration, and participation in social and life roles

For the Stroke prevention competency, most of the participants rated themselves as advanced beginners, except for item Utilize education resources available to support teaching on secondary prevention, where most participants rated themselves as competent (7/25, 28%). For Transitions and community integration, most participants rated themselves as advanced beginners. For Participation in social and life roles, most of the participants rated themselves as advanced beginners for all the competency items (Table 7).

**Table 7:** Ratings on stroke prevention, transitions and community integration, and participation in social and life roles

Competency	Domain items	Frequency (proportion), N=25				
		Novice, n (%)	Advanced beginner, n (%)	Competent, n (%)	Proficient, n (%)	Expert, n (%)
Stroke prevention	Identify the warning signs of stroke and transient ischemic attack	2 (8)	10 (40)	10 (40)	3 (12)	0
	Identify and provide examples of the modifiable and non-modifiable risk factors for stroke	3 (12)	11 (44)	7 (28)	3 (12)	1 (4)
	Identify modifiable risk factor management strategies for the reduction of secondary or recurrent stroke	3 (12)	10 (40)	9 (36)	3 (12)	0
	Utilize education resources available to support teaching on secondary prevention	6 (24)	8 (8)	7 (28)	2 (8)	2 (8)
	Effectively communicate the recommendations and appropriate referrals, services, and resources to the persons with stroke, families/caregivers and the interprofessional team	4 (16)	9 (36)	6 (24)	4 (16)	2 (8)
19. Transitions and community integration	Develop individualized care plans with persons with stroke and their families that are person-centered and culturally appropriate	4 (16)	12 (48)	5 (20)	3 (12)	1 (4)
	Provide comprehensive and relevant information in a timely manner to ensure seamless transitions and continuity of care	2 (8)	9 (36)	8 (32)	4 (16)	2 (8)
	Describe the process for accessing post-acute stroke rehabilitation services	5 (20)	9 (36)	7 (28)	4 (16)	0
	Identify the impacts of transitions on persons living with stroke and understand how to prepare for transitions between care	3 (12)	13 (52)	5 (20)	4 (16)	0
	Identify available transitional/discharge resources and services for post-stroke care and recovery relevant to nursing	5 (20)	11 (44)	6 (24)	2 (8)	1 (4)
	Describe the impact of stroke on the caregiver and family members post-stroke	2 (8)	10 (40)	9 (36)	3 (12)	1 (4)
Participation in social and life roles	Summarize the impact of participating in social and life roles post-stroke - sexuality	7 (28)	9 (36)	6 (24)	2 (8)	1 (4)
	Summarize the impact of participating in social and life roles post-stroke - relationships	4 (16)	10 (40)	7 (28)	2 (8)	2 (8)
	Summarize the impact of participating in social and life roles post-stroke - vacation	3 (12)	13 (52)	6 (24)	1 (4)	2 (8)
	Summarize the impact of participating in social and life roles post-stroke - leisure activities	5 (20)	9 (36)	6 (24)	4 (16)	1 (4)
	Summarize the impact of participating in social and life roles post-stroke - driving/transport	6 (24)	9 (36)	5 (20)	2 (8)	3 (12)
	Screen for the impact on participation in social and life roles and make appropriate referrals	2 (8)	11 (44)	6 (24)	3 (12)	3 (12)
	Select and complete the most appropriate assessment(s)	7 (28)	9 (36)	6 (24)	2 (8)	1 (4)
	Interpret assessment results, identify/implement appropriate	5 (20)	8 (32)	8 (32)	2 (8)	2 (8)
	Demonstrate communication skills and knowledge to address physical, emotional, and cognitive impact on social and life roles	6 (24)	8 (32)	9 (36)	1 (4)	1 (4)
	Effectively communicate the recommendations and appropriate referrals	6 (24)	8 (32)	7 (28)	3 (12)	1 (4)

Disposition and reporting

For disposition and reporting, the majority of the participants were advanced beginners, followed by the competent participants. There was no expert participant for two competencies, Advance care planning and Reporting and performance. For the rest of the competencies, at least one or two participants reported to be experts in the different competencies and domain items. These included Patient, family, and caregiver education, where for all items, very few participants were proficient (12% and below). For item Demonstrate accurate documentation of stroke-specific data, 16 (64%) rated themselves as novice or advanced beginners (Table 8).

**Table 8:** Ratings on disposition and reporting

Competency	Domain items	Frequency (proportion), N=25				
		Novice, n (%)	Advanced beginner, n (%)	Competent, n (%)	Proficient, n (%)	Expert, n (%)
Advanced care planning	Recognize when to reassess goals of care (i.e., change in a patient’s health status)	4 (16)	7 (28)	8 (32)	6 (24)	0 (0)
Palliative and end-of-life care	Describe the key content to be addressed to support comfort and quality of life	3 (12)	13 (52)	4 (16)	4 (16)	1 (4)
	Demonstrate a palliative approach to care to support basic symptom	1 (4)	13 (52)	8 (32)	2 (8)	1 (4)
	Identify when a palliative and end-of-life discussion is warranted	3 (12)	11 (44)	7 (28)	3 (12)	1 (4)
Patient, family, and caregiver education	Conduct an assessment of individualized learning needs and goals through stages of care in collaboration with the person with stroke and the interprofessional team	4 (16)	9 (36)	9 (36)	2 (8)	1 (4)
	Develop individualized education plan based on the assessment of learning needs and goals	3 (12)	10 (40)	7 (28)	3 (12)	2 (8)
	Identify strategies to promote self-management (e.g., motivational	4 (16)	10 (40)	9 (36)	1 (4)	1 (4)
	Identify appropriate ways to communicate and support retention of information to the persons with stroke, families/caregivers, and the interprofessional team	4 (16)	12 (48)	5 (20)	2 (8)	2 (8)
Reporting and performance	Demonstrate accurate documentation of stroke-specific data elements	6 (24)	10 (40)	5 (20)	4 (16)	0

## Discussion

In this study, we described the priorities for stroke competence-based training among nurses taking care of people with stroke at a large hospital in southwestern Uganda. We observed that most participants were diploma holders, and only 8% of the participants had more than 15 years of experience. For most of the competencies and the corresponding items, most participants rated themselves as novice or advanced beginners. These competencies were from the Stroke Core Competency Framework and included Anatomy, physiology, and hyperacute care for stroke; Stroke types, dysphagia, and nutrition and hydration for persons with stroke; Oral care, activity, and communication; Physical function, continence, post-stroke pain and spasticity; Stroke prevention, transitions and community integration, and participation in social and life roles; and Disposition and reporting.

Almost all participants reported being either novice or advanced beginners in stroke core competencies, despite the level of training reflecting a gap in the training of nurses at all levels. The current nursing training programs do not cover adequate stroke nursing care competencies. We also observed that more than half of the participants were diploma holders and below, and a few were degree holders. Diploma nursing education takes a maximum of three years, whereas a degree training takes a minimum of four years of advanced management, research, and patient care. This presents an opportunity for the nurses to upgrade to higher education, and the national curriculum designers to develop diploma and certificate programs that include stroke core competencies in their content. Diploma and certificate holder nurses are the largest section of the nurses employed [19]. This is alarming in view of the increasing stroke incidences in both the young and older populations in Uganda [20].

Although all participants had been in service for 1-15 years, they were still novice or advanced beginners in stroke core competencies. This asserts the observation by Benner that experience is not the mere passage of time or longevity; it is the refinement of preconceived notions and theory by encountering many actual practical situations that add nuances or shades of differences to theory [16]. In addition to inadequate pre-service training, there is no arrangement for continuous professional development (CPD) for nurses in service to impart stroke care competencies [21]. This leaves a big gap in nurses’ competencies in stroke care.

We also observed that the mean age of the participants was 31.8 years. This resonates with the general demographics of Uganda’s population and existing human resources management research that suggests that younger employees who are provided with training and development opportunities tend to show higher levels of engagement and retention within an organization [22]. Also, this provides an opportunity for healthcare managers in Uganda to provide education interventions to mitigate this gap in competencies for stroke care. In the long run, this in-service training may motivate nurses to improve skills, with health systems strengthening, thus improving patient outcomes as observed previously [23].

Our study had some limitations as well. This study recruited a relatively small number of participants; therefore, we did not perform any inferential statistics. However, this was a census in which we sampled all nurses engaged in stroke care at the hospital at the time of the study. Also, we considered only one facility, which is the only hospital that provides stroke care in southwestern Uganda. The single-center design limits generalizability, although results are useful to other hospitals in low-resource settings. The SCCF collected self-reported competencies; although this allows us to make meaningful conclusions about the exact picture of nurses’ competencies in stroke care, we acknowledge that this allowed for reporting bias. To our knowledge, this is the first study of its kind in southwestern Uganda; therefore, we have only described the self-rated competencies, and hence, there is a need for objective performance assessment. The competencies were assessed using self-rated scores rather than an objective performance assessment of the stroke care core competency; thus, individual biases cannot be considered. However, we used a standard, validated tool that improved the credibility of the assessment.

## Conclusions

Overall, nurses involved in providing care for people with stroke at Mbarara Regional Referral Hospital are novice or advanced beginners. This study provides a context‑specific assessment of nurses’ stroke care competencies in southwestern Uganda. These results and similar future studies may influence the development and design of context-specific and competency-based continuing education for stroke nurses. This will foster the implementation of best practices in stroke care and designing stroke-based training modules for nurses to achieve optimal competencies.
